# External validation of the BEST-J score and a new risk prediction model for ESD delayed bleeding in patients with early gastric cancer

**DOI:** 10.1186/s12876-022-02273-2

**Published:** 2022-04-20

**Authors:** Jiaxu Wang, Shanshan Wu, Jie Xing, Peng Li, Shutian Zhang, Xiujing Sun

**Affiliations:** Department of Gastroenterology, Beijing Friendship Hospital, Capital Medical University, National Clinical Research Center for Digestive Diseases, Beijing Digestive Disease Center, Faculty of Gastroenterology of Capital Medical University, Beijing Key Laboratory for Precancerous Lesion of Digestive Diseases, Beijing, 100050 China

**Keywords:** Delayed bleeding, Early gastric cancer, Endoscopic submucosal dissection, Prediction model, Risk factors

## Abstract

**Background:**

Delayed bleeding is an important adverse event after gastric endoscopic submucosal dissection (ESD). We aimed to externally validate the Bleeding after ESD Trend from Japan (BEST-J) score and subsequently develop a risk prediction model for bleeding in Chinese patients with early gastric cancer (EGC) after ESD.

**Methods:**

The clinical data of patients who underwent ESD for EGC in Beijing Friendship Hospital from June 2013 to December 2019 were collected retrospectively. The BEST-J score was evaluated according to the clinical data. Through univariate and multivariate logistic regression analyses of the clinical data, the factors affecting delayed bleeding were identified, and a new risk prediction model for bleeding was established. Receiver operating characteristic (ROC) curves were used to evaluate the predictive value of the two prediction models.

**Results:**

A total of 444 patients with EGC undergoing ESD were included, of whom 27 patients had delayed bleeding (6.1%). Multivariate logistic regression analysis showed that a history of smoking (P = 0.029), tumor size > 20 mm (P = 0.022), intraoperative use of hemoclips (P = 0.025), resection of multiple tumors (P = 0.027), and prolongation of activated partial thromboplastin time (APTT) (P = 0.020) were independent influencing factors for delayed bleeding. ROC curve analysis showed that the areas under the curves (AUCs) were different between the BEST-J score and the newly built prediction model (0.624 vs. 0.749, P = 0.012).

**Conclusions:**

The BEST-J score has moderately good discrimination for Chinese patients with EGC. However, for patients with EGC without severe comorbidities, the new risk prediction model may predict delayed bleeding better than the BEST-J score.

**Supplementary Information:**

The online version contains supplementary material available at 10.1186/s12876-022-02273-2.

## Background

In recent years, with the advancement of endoscopic technology, endoscopic submucosal dissection (ESD) has increasingly become the main choice for early gastric cancer (EGC) on account of its lower incidence of adverse events, association with a shorter hospital stay, lower cost and higher quality of life than surgery [1–3]. However, ESD requires high technology, and adverse events can follow. The most common adverse event of gastric ESD is bleeding [4, 5]. Immediate intraoperative bleeding is easily identified during ESD surgery and can be treated by endoscopy in most cases. Delayed bleeding may occur a few days after ESD and occasionally even after discharge. If endoscopists cannot detect bleeding in time, some cases may progress to hemorrhagic shock or even death. Therefore, endoscopists should fully evaluate the risk of delayed bleeding so that patients at high risk of bleeding can receive enough attention.

Clinical prediction models have been used in a number of fields to estimate the value of therapy and prognosis of individual patients [6–8], but there are few reports on prediction models for bleeding after ESD for EGC. A national multicenter study in Japan provided a prediction model for bleeding risk after ESD [Bleeding after ESD Trend from Japan (BEST-J) score] [9], in which 10 variables (warfarin, direct oral anticoagulant, chronic kidney disease with hemodialysis, P2Y12 receptor antagonist, aspirin, cilostazol, tumor size > 30 mm, lower-third in tumor location, presence of multiple tumors and interruption of each kind of antithrombotic agent) were identified. However, this model lacks cohort verification in other countries and regions, and its feasibility and generalization still need to be considered. The purpose of our study was to verify the applicability of the BEST-J score to Chinese patients. Furthermore, we explored a more suitable risk prediction model for bleeding after ESD in Chinese patients with EGC.

## Methods

### Patients

**Fig. 1:**
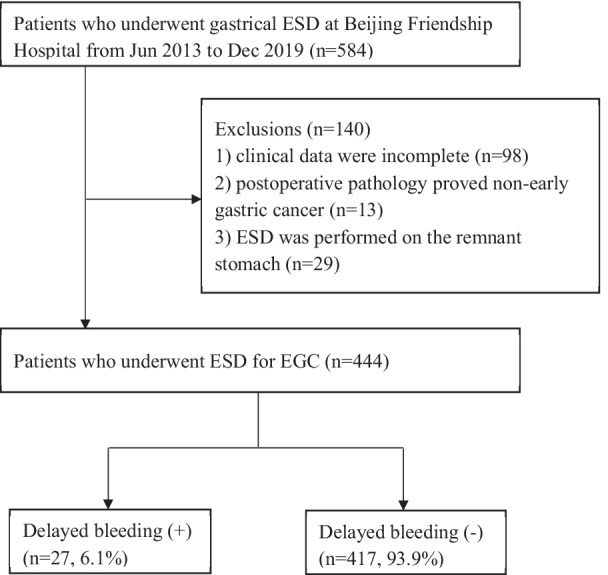
The flowchart of patient enrollment. EGC, early gastric cancer; ESD, endoscopic submucosal dissection

The study population consisted of adult patients who underwent ESD for EGC in Beijing Friendship Hospital from June 2013 to December 2019. Patients were excluded if they met the following criteria: (1) clinical data were incomplete, (2) postoperative pathology proved nonearly gastric cancer, or (3) ESD was performed on the remnant stomach.(Fig. 1) This study was approved by the ethics committee of Beijing Friendship Hospital (no. 2020-P2-290–01).

All patients taking antiplatelet and antithrombotic drugs were asked to stop the medications for at least 7 days before ESD. For patients with a high thromboembolic risk, bridging therapy using heparin was performed. For patients using dual antiplatelet or antithrombotic medications, clopidogrel or warfarin was stopped without interruption of low-dose aspirin. All antiplatelet or antithrombotic drugs were restarted 1–2 days after ESD.

### ESD procedure

ESD was performed according to a standard ESD procedure [10]. Briefly, the procedure consisted of the following steps: (1) marking around the lesion, (2) submucosal injection, (3) mucosal incision outside of the marked region, (4) submucosal dissection, and (5) retrieval of the specimen. After lesion removal, preventive coagulation was performed on all visible vessels.

ESD was performed using an IT Knife2 (KD-611L; Olympus, Tokyo, Japan) or a Dual Knife (KD-650L; Olympus). GIF-H260Z (Olympus) was used for assessing the lesion range and preoperative marking. For ESD, we used GIF-Q260J (Olympus) and a high-frequency generator (VIO300D; ERBE, Tubingen, Germany). A Coagrasper (FD-410LR; Olympus) was used for blood vessel cauterization during ESD or after ESD. ESD operations were performed by expert endoscopists who have performed more than 100 gastric ESD procedures [11].

### Definition

Delayed bleeding, or bleeding after ESD, was generally defined as any clinical evidence of bleeding after ESD, such as hematemesis, melena, hemodynamic deterioration or a decrease of > 2 g/dL in hemoglobin level that required endoscopic hemostasis [10, 12].

If multiple tumors were present, the location and macroscopic type of the largest tumor were recorded.

Tumor location was classified as the lower-third, middle-third, or upper-third of the stomach [13].

The procedure time was calculated from marking the lesion to completion of preventive coagulation of all visible vessels after lesion removal.

The depth of tumor invasion is recorded as the deepest layer that the cancer has infiltrated. Furthermore, for cancers invading the submucosa, we measured the distance (in μm) from the lower margin of the muscularis mucosa to the deepest part of the invading cancer. If this measurement depth was < 500 μm, we assessed and classified the invasion as SM1 (or T1b1), and if the depth was ≥ 500 μm, the invasion was classified as SM2 (or T1b2) [14].

The intraoperative use of hemoclips was considered the use of any metal clip [including harmony clip (ROCC-D-26–195-C; Micro-Tech, Nanjing, China), Long clip (HX-610-135L; Olympus), etc.] during the procedure. Endoscopists decide whether or not to use hemoclips during the procedure according to different wound conditions and their assessment of delayed bleeding risk under gastroscopy.

Absolute indications for ESD are (1) UL0 cT1a differentiated-type carcinomas with a long diameter greater than 2 cm; (2) UL1 cT1a differentiated-type carcinomas with a long diameter measuring 3 cm or less; and (3) UL0 cT1a undifferentiated-type carcinomas with a long diameter 2 cm or less [15].

Curative resection for ESD requires all of the following [15]: en bloc resection, long diameter ≤ 2 cm, differentiated type, pT1a, UL0, HM0, VM0, Ly0, V0.

Patients’ blood examinations results, including the hemoglobin value, platelet value, prothrombin time (PT), prothrombin time activity [PT(A)], international standardized ratio (INR) and activated partial thromboplastin time (APTT), were obtained 1–2 days before ESD.

### Statistical analysis

SPSS 20.0 and SAS 9.4 was used for data analysis. Categorical data are expressed as absolute numbers with percentages, and the chi-square test or Fisher’s exact test was used for comparisons. Continuous data are expressed as the mean ± SD, and an independent sample t-test was used for comparisons. Univariate analysis was carried out by using delayed bleeding as the dependent variable. Incorporating the associated predictive factors obtained from the literature review, variables with a P value < 0.1 in univariate analyses were selected for inclusion in the univariate logistic regression analysis. Multivariate logistic regression analysis was performed to assess the factors associated with bleeding after ESD in patients with EGC and to establish a risk prediction model for bleeding. Receiver operating characteristic (ROC) curves were used to evaluate the discrimination of the BEST-J score and the newly established risk prediction model. The areas under the curves (AUCs) were compared between different models with the Delong test by MedCalculator software. A P value < 0.05 was considered statistically significant.

## Results

### Comparison of the basic characteristics of the patients

A total of 444 patients with EGC undergoing ESD were divided into two groups: the delayed bleeding group (n = 27) (6.1%) and the nonbleeding group (n = 417) (93.9%). In terms of the basic characteristics, there were no significant differences in age, sex, hypertension, coronary heart disease, diabetes, use of antithrombotic medication (including anticoagulants and antiplatelet drugs), antidepressants, proprietary Chinese medicine, history of drinking or family history of digestive tract tumors between the nonbleeding group and delayed bleeding group (P > 0.05). There was a significant difference in the distribution of smoking history between the two groups (P = 0.044). See Table 1 for details.

**Table 1:** Comparison of the basic characteristics of patients with EGC between the delayed bleeding group and the nonbleeding group

	Nonbleeding group (n = 417)	Delayed bleeding group (n = 27)	χ^2^ or t value	*P* value
Age (years, mean ± SD)	63.08 ± 9.317	63.00 ± 12.175	0.041	0.135
Sex [n (%)]			0.177	0.674
Male	293 (70.3)	20 (74.3)		
Female	124 (29.7)	7 (25.9)		
Hypertension [n (%)]			0.073	0.787
No	258 (61.9)	16 (59.3)		
Yes	159 (38.1)	11 (40.7)		
Coronary heart disease [n (%)]			NA	1.000
No	376 (90.2)	25 (92.6)		
Yes	41 (9.8)	2 (7.4)		
Diabetes [n (%)]			NA	0.444
No	341 (81.8)	24 (88.9)		
Yes	76 (18.2)	3 (11.1)		
Anticoagulant medication history [n (%)]			NA	0.172
No	415 (99.5)	26 (96.3)		
Yes	2 (0.5)	1 (0.2)		
Antiplatelet medication history [n (%)]			NA	1.000
No	376 (90.2)	25 (92.6)		
Yes	41 (9.8)	2 (7.4)		
Antidepressant medication history [n (%)]			NA	0.223
No	414 (99.3)	26 (96.3)		
Yes	3 (0.7)	1 (0.2)		
Proprietary Chinese medicine history [n (%)]			NA	0.614
No	402 (96.4)	27 (100.0)		
Yes	15 (3.6)	0 (0.0)		
Smoking [n (%)]			4.072	0.044*
No	252 (60.4)	11 (40.7)		
Yes	165 (39.6)	16 (59.3)		
Drinking [n (%)]			1.904	0.168
No	271 (65.0)	14 (51.9)		
Yes	146 (35.0)	13 (48.1)		
Family history of digestive tract tumors [n (%)]			1.188	0.276
No	317 (76.0)	23 (85.2)		
Yes	100 (24.0)	4 (14.8)		

EGC, early gastric cancer; NA, not available due to Fisher’s exact test; SD, standard deviation

*P < 0.05

### Comparison of the clinical characteristics of the patients

The clinical characteristics of the patients are shown in Table 2. There were no significant differences in procedure duration, tumor location, number of resected specimens, lifting sign, resection type, procedure time, macroscopic type, tumor differentiation, pathological classification, depth of tumor invasion, vertical margin, horizontal margin, lymphatic invasion, vascular invasion, complete resection, resection classification or absolute indication between the nonbleeding group and the delayed bleeding group (P > 0.05). There were significant differences in tumor size, the intraoperative use of hemoclips and resection of multiple tumors between the two groups (P < 0.05).

**Table 2:** Comparison of the clinical characteristics of patients with EGC between the delayed bleeding group and the nonbleeding group

	Nonbleeding group (n = 417)	Delayed bleeding group (n = 27)	χ^2^ or t value	*P* value
Procedure duration (min, mean ± SD)	87.09 ± 72.40	79.70 ± 49.44	0.260	0.260
Procedure duration [n (%)]			< 0.001	0.994
≤ 60 min	185 (44.4)	12 (44.4)		
> 60 min	232 (55.6)	15 (55.6)		
Tumor size [n (%)]			5.790	0.016*
≤ 20 mm	293 (70.3)	13 (48.1)		
> 20 mm	124 (29.7)	14 (51.9)		
Tumor location [n (%)]			NA	0.129
Upper	127 (30.5)	4 (14.8)		
Medial	27 (6.5)	3 (11.1)		
Lower	263 (63.1)	20 (74.1)		
Number of resected specimen [n (%)]			NA	0.196
Single	374 (89.7)	22 (81.5)		
Multiple	43 (10.3)	5 (18.5)		
Lifting sign [n (%)]			0.012	0.914
Positive	328 (78.7)	21 (77.8)		
Poor	89 (21.3)	6 (22.2)		
Intraoperative use of hemoclips [n (%)]			5.209	0.022*
No	265 (63.5)	23 (85.2)		
Yes	152 (36.5)	4 (14.8)		
Resection type [n (%)]			NA	0.643
Piecemeal	21 (5.0)	2 (7.4)		
En bloc	396 (95.0)	25 (92.6)		
Procedure time [n (%)]			0.652	0.419
Morning	234 (56.1)	13 (48.1)		
Afternoon	183 (43.9)	14 (51.9)		
Macroscopic type [n (%)]			NA	1.000
Type 0-I	19 (4.6)	1 (3.7)		
Type 0-II	396 (95.0)	26 (96.3)		
Type 0-III	2 (0.5)	0 (0.0)		
Tumor differentiation [n (%)]			NA	0.721
Differentiated	381 (91.4)	24 (88.9)		
Undifferentiated	36 (8.6)	3 (11.1)		
Histological classification [n (%)]			NA	0.676
LGIN	6 (1.4)	0 (0.0)		
HGIN	45 (10.8)	4 (14.8)		
Gastric cancer	366 (87.8)	23 (85.2)		
Depth of tumor invasion [n (%)]			NA	0.896
M	367 (88.0)	24 (88.9)		
SM1	34 (8.2)	3 (11.1)		
SM2	16 (3.8)	0 (0.0)		
Vertical margin [n (%)]			NA	0.300
Negative	401 (96.2)	25 (92.6)		
Positive	16 (3.8)	2 (7.4)		
Horizontal margin [n (%)]			NA	0.077
Negative	403 (96.6)	24 (88.9)		
Positive	14 (3.4)	3 (11.1)		
Lymphatic invasion [n (%)]			NA	0.324
No	400 (95.9)	25 (92.6)		
Yes	17 (4.1)	2 (7.4)		
Venous invasion [n (%)]			< 0.001	1.000
No	399 (95.7)	26 (96.3)		
Yes	18 (4.3)	1 (3.7)		
Complete resection [n (%)]			NA	0.204
No	23 (5.5)	3 (11.1)		
Yes	394 (94.5)	24 (88.9)		
Resection classification [n (%)]			NA	0.120
Curative resection	243 (58.3)	11 (40.7)		
Curative resection for expanded indications	90 (21.6)	10 (37.0)		
Noncurative resection	84 (20.1)	6 (22.2)		
Multiple tumors [n (%)]			NA	0.036*
No	390 (93.5)	22 (81.5)		
Yes	27 (6.5)	5 (18.5)		
Absolute indication [n (%)]			3.137	0.077
Yes	257 (61.6)	12 (44.4)		
No	160 (38.4)	15 (55.6)		

EGC, early gastric cancer; HGIN, high-grade intraepithelial neoplasia; LGIN, low-grade intraepithelial neoplasia; M, confined to the mucosa; NA, not available due to Fisher’s exact test; SD, standard deviation; SM1, submucosal invasion < 500 µm from the muscularis mucosa; SM2, submucosal invasion ≥ 500 µm from the muscularis mucosa

*P < 0.05

### Comparison of patients’ laboratory results

The preoperative laboratory results of the patients are shown in Table 3. There were no significant differences in hemoglobin value, platelet value, PT, PT(A) or INR between the delayed bleeding group and the nonbleeding group (P > 0.05). There was a significant difference in APTT between the two groups (P = 0.009).

**Table 3:** Comparison of the laboratory results of patients with EGC between the delayed bleeding group and the nonbleeding group

	Nonbleeding group (n = 417)	Delayed bleeding group (n = 27)	*P* value
Hemoglobin value [n (%)]			0.061
≥ 90 g/L	417 (100.0)	26 (96.3)	
< 90 g/L	0 (0.0)	1 (3.7)	
Platelet value [n (%)]			1.000
≥ 125 × 10^9^/L	389 (93.3)	26 (96.3)	
< 125 × 10^9^/L	28 (6.7)	1 (3.7)	
PT [n (%)]			0.397
≤ 13.5 s	410 (98.3)	26 (96.3)	
> 13.5 s	7 (1.7)	1 (3.7)	
PT(A) [n (%)]			0.650
≥ 80%	395 (94.7)	25 (92.6)	
< 80%	22 (5.3)	2 (7.4)	
INR [n (%)]			0.315
≤ 1.2	412 (98.8)	26 (96.3)	
> 1.2	5 (1.2)	1 (3.7)	
Prolongation of APTT [n (%)]			0.009*
No	406 (97.4)	23 (85.2)	
Yes	11 (2.6)	4 (14.8)	

APTT, activated partial thromboplastin time; EGC, early gastric cancer; INR, international standardized ratio; PT, prothrombin time; PT(A), prothrombin time activity

*P < 0.05

### External validation of the BEST-J score

**Fig. 2:**
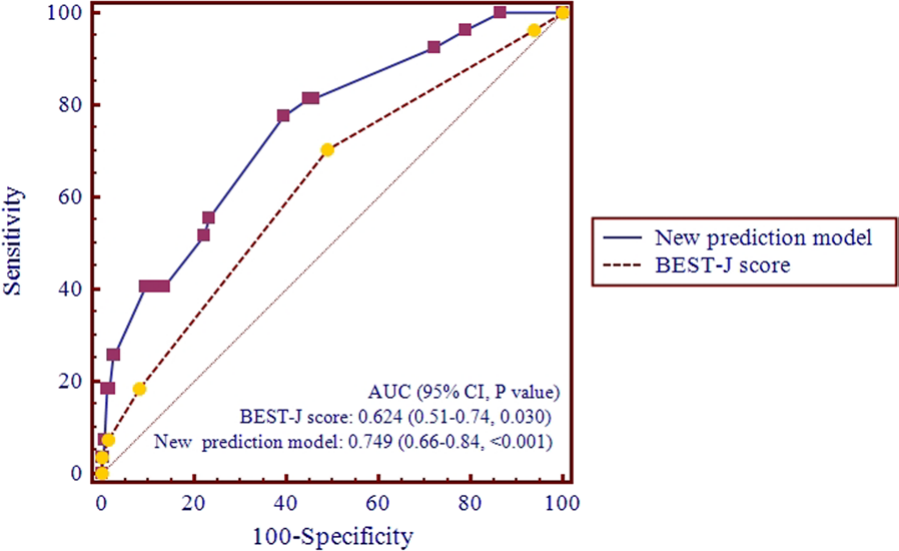
ROC curves and AUCs of the new prediction model and the BEST-J score. AUC, area under the curve; BEST-J, bleeding after ESD trend from Japan; CI, confidence interval; ROC, receiver operating characteristic

There were significant differences in the distribution of bleeding risk scores and risk classification for EGC in the validation cohort for the BEST-J score (P < 0.05) (Table 4). The AUC in the validation cohort was 0.624 (95% CI: 0.514–0.735; P = 0.030), which shows that the BEST-J score has moderately good discrimination for Chinese patients with EGC (Fig. 2). The comparison between the derivation and validation cohorts is detailed in Additional file 1 and Additional file 2.

**Table 4:** Comparison of BEST-J scores of patients with EGC between the delayed bleeding group and the nonbleeding group

	Nonbleeding group (n = 417)	Delayed bleeding group (n = 27)	*P* value
Total scores (points, mean ± SD)	1.53 ± 0.778	1.96 ± 1.018	0.006*
Total scores [n (%)]			0.047*
0	25 (6.0)	1 (3.7)	
1	187 (44.8)	7 (25.9)	
2	171 (41.0)	14 (51.9)	
3	28 (6.7)	3 (11.1)	
4	5 (1.2)	1 (3.7)	
5	1 (0.2)	1 (3.7)	
Risk category [n (%)]			0.020*
Low-risk^†^	212 (50.8)	8 (29.6)	
Intermediate-risk^†^	171 (41.0)	14 (51.9)	
High-risk^†^	33 (7.9)	4 (14.8)	
Very high-risk^†^	1 (0.2)	1 (3.7)	

BEST-J, bleeding after ESD trend from Japan; EGC, early gastric cancer; SD, standard deviation

^†^Low-risk (total points = 0 or 1), intermediate-risk (total points = 2), high-risk (total points = 3 or 4), and very high-risk (total points ≥ 5)

*P < 0.05

### Univariate and multivariate logistic regression analysis

The variables with a *P* value < 0.1 in univariate analysis were screened out for univariate logistic regression analysis. There were significant differences in smoking history, tumor size, intraoperative use of hemoclips, resection of multiple tumors and prolongation of APTT (*P* < 0.05). The above factors were included in the logistic regression model for multivariate analysis. Smoking history (yes = 1; no = 0), tumor size (> 20 mm = 1; ≤ 20 mm = 0), intraoperative use of hemoclips (yes = 1; no = 0), resection of multiple tumors (yes = 1; no = 0), and prolongation of APTT (yes = 1; no = 0) were independent variables, and postoperative outcome was a dependent variable (bleeding = 1; nonbleeding = 0). Multivariate logistic regression analysis showed that a history of smoking (P = 0.029), tumor size > 20 mm (P = 0.022), intraoperative use of hemoclips (P = 0.025), resection of multiple tumors (P = 0.027), and prolongation of APTT (P = 0.020) were independent influencing factors for delayed bleeding (Table 5).

**Table 5:** Univariate and multivariate logistic regression analysis of delayed bleeding in patients with EGC

	Univariate logistic regression				Multivariate logistic regression				
	*OR* value	95% *CI*	*P* value		*OR* value		95% *CI*	*P* value	
Smoking									
Yes	2.221	1.006–4.906		0.048	2.564	1.103–5.963			0.029*
No	1				1				
Tumor size									
> 20 mm	2.545	1.162–5.571		0.019	2.630	1.148–6.029			0.022*
≤ 20 mm	1				1				
Intraoperative use of hemoclips									
Yes	0.303	0.103–0.893		0.030	0.282	0.093–0.854			0.025*
No	1				1				
Horizontal margin									
Negative	3.598	0.968–13.378		0.056	NA	NA			NA
Positive	1								
Multiple tumors									
Yes	3.283	1.153–9.348		0.026	3.621	1.161–11.291			0.027*
No	1				1				
Absolute indication									
Yes	0.498	0.227–1.091		0.082	NA	NA			NA
No	1								
Prolongation of APTT									
Yes	6.419	1.897–21.722		0.003	4.923	1.282–18.907			0.020*
No	1				1				

APTT, activated partial thromboplastin time; CI, confidence interval; EGC, early gastric cancer; NA means that this factor was not included in the multivariate logistic regression analysis; OR, odds ratio

*P < 0.05

### Establishment of the new risk prediction model

The new risk prediction model for bleeding was established based on the results of the multivariate logistic regression analysis: P = e^X^/(1 + e^X^) × 100, where ‘e’ is the natural logarithm, and X = − 3.548 + 0.942 × (a history of smoking) − 1.265 × (intraoperative use of hemoclips) + 1.287 × (resection of multiple tumors) + 0.967 × (tumor size > 20 mm) + 1.594 × (prolongation of APTT) (Table 6). The AUC of the prediction model was 0.749 (*P* < 0.001) (Fig. 2).

**Table 6:** Distribution of risk scores for bleeding after ESD for EGC according to the new risk prediction model

Total points	Patients (n = 444)	Bleeding (n = 27)	Rate of bleeding (95% CI) (%)
1–2	117	2	1.71% (0.002–0.060)
3–4	117	3	2.56% (0.005–0.073)
5–9	157	11	7.01% (0.036–0.122)
12–17	35	4	11.43% (0.032–0.267)
21–57	18	7	38.89% (0.173–0.643)

CI, confidence interval; EGC, early gastric cancer; ESD, endoscopic submucosal dissection

### Comparison of predictive values between the BEST-J score and the new risk prediction model

The AUCs were significantly different between the BEST-J score and the newly built prediction model (AUC difference = 0.125, P = 0.012) (Fig. 2). The new prediction model can evaluate the risk of bleeding after ESD in Chinese patients with EGC better than the BEST-J score.

## Discussion

In this study, we explored a large number of factors related to bleeding after ESD and found five independent factors related to delayed bleeding: a history of smoking, tumor size > 20 mm, intraoperative use of hemoclips, resection of multiple tumors and prolongation of APTT. Among them, the intraoperative use of hemoclips is a protective factor against delayed bleeding in ESD, and the others are risk factors. Based on the selected influencing factors, we developed a risk score model to predict the risk of bleeding after ESD in patients with EGC.

According to the WHO definition [16], smokers are defined as patients who have smoked continuously or cumulatively for more than 6 months. In a controlled study, Ying Shi et al. [17] found that smoking increased the risk of short-term bleeding after coronary intervention in patients with coronary heart disease (*P* = 0.03). A national analysis in the United States also concluded that smoking was associated with a higher risk of surgical bleeding (OR = 1.06, 95% CI 1.05–1.07) [18]. In our study, smokers had a higher risk of delayed bleeding than non-smokers, which is consistent with the above research results. Mechanistically, smoking increases the recruitment of inflammatory cells to the vessel wall and triggers the release of a variety of oxidative molecules, leading to damage to the walls of blood vessels [19, 20]. Damage to the vessel wall could in turn make the vessels more vulnerable and more likely to rupture and bleed.

Previous studies have shown that tumor size is the most important predictor of bleeding after ESD for EGC [21–24]. Interestingly, previous studies have reported different tumor sizes that affect delayed bleeding after ESD. In a retrospective analysis of 418 elderly patients with EGC who underwent ESD, there was a significant correlation between a tumor size ≥ 4 cm and delayed bleeding (P = 0.026) [22]. A recent Chinese study [21] also showed that a tumor size ≥ 4 cm (95% CI 0.721–6.345, P = 0.013) was the main risk factor for delayed bleeding. However, a meta-analysis of 74 articles [23] showed that a tumor size > 20 mm (OR = 2.70) was an important factor for postoperative bleeding. Another study of 210 patients [24] showed that a lesion diameter > 20 mm (95% CI 1.047–2.064, P < 0.05) was an independent risk factor for delayed bleeding. Combined with our findings, it is considered that a tumor size > 20 mm is more valuable in evaluating bleeding after ESD. Theoretically, the larger the lesion is, the higher the risk of delayed bleeding because large lesions will form large post-ESD ulcers, which require more time for mucosal regeneration and healing. However, we did not observe this trend in terms of the size of resected specimens. The reason may be that the vascular supply to the invasive part of the tumor is larger than that to normal tissue. Larger sample size studies are needed to verify this.

The intraoperative use of hemoclips was the only protective factor for delayed bleeding in our study. The ESD technique may result in a large mucosal defect at the resection site. Closure of the defect with endoscopic hemoclips leaves no submucosal tissue or muscularis mucosa exposed to the polluted environment, which can accelerate wound healing and reduce the rate of bleeding and perforation [25, 26]. In addition, the mechanical force generated when the hemoclips are closed can compress the bleeding blood vessels and surrounding tissues so as to achieve the effect of blocking blood flow [27]. Liu Tao et al. pointed out that the preventive use of hemoclips after gastric polypectomy can effectively stop bleeding and prevent delayed bleeding [27]. Azumi et al. divided the patients with gastric ESD into observation group and control group according to whether preventive hemoclip treatment was performed. By comparing the rate of delayed bleeding between the two groups, it was found that the rate of delayed bleeding decreased significantly in the observation group [28]. The results of these studies are consistent with our study, indicating that intraoperative use of hemoclips can reduce the rate of delayed bleeding after operation. However, the quantity and cost-effectiveness of hemoclips need to be further studied.

Previous studies have shown the guiding significance of APTT in predicting bleeding. Guorong Zhang et al. [29] found that monitoring blood coagulation function indexes such as APTT is of great significance for the prevention and treatment of bleeding in premature infants. Hasegawa et al. [30] pointed out that the analysis of the APTT waveform may be useful for the prediction of the risk of massive bleeding after orthopedic surgery. To our knowledge, this study found for the first time that prolonged APTT is associated with an increased risk of bleeding after ESD. The normal APTT levels are 21–34 s in our study. Due to differences in the definition of a healthy population, technology, evaluation method, instruments and actual procedure in different regions, the reference range of APTT can vary [31]. Therefore, no specific value of APTT was set in this study, but the prolongation of APTT was taken as the influencing factor. APTT is the most commonly used and sensitive screening index to reflect the endogenous coagulation system. It is widely used in the diagnosis, treatment and monitoring of coagulation disorders, bleeding and thrombotic diseases. Many diseases, such as acute and chronic liver disease, hepatolithiasis, rheumatic immune disease, hypercoagulable state, thrombotic disease, nephropathy, etc. can either lead to the consumption of a large number of coagulation factors or affect the release of coagulation factors, resulting in prolonged APTT time. APTT value is simple and easy to obtain before operation. Its prolongation reflects the possibility of increased risk of delayed bleeding, which may be of guiding value in the prevention of bleeding after ESD.

Although the BEST-J score showed good overall performance and calibration in the study from Wakuhatta et al. [9] and can be used as a simple assistant tool for clinical decision-making in routine practice, the model was established based on an analysis of Japanese patients. When the model is applied to Chinese patients, its ability to predict the risk of postoperative bleeding is reduced. The reasons may be as follows: 1. different countries have different standards for the diagnosis and treatment of ESD. The Japan Gastroenterological Endoscopy Society (JGES) guidelines recommend that aspirin treatment should be continued in patients receiving aspirin alone or aspirin in combination with thienopyridine even if there is a high risk of bleeding from endoscopic gastroenterological procedures [32]. In contrast, Chinese experts agree that anticoagulants and antiplatelet drugs should be discontinued for 5–7 days prior to the procedure [33]. This is also an important reason for the lack of patients at high risk and very high risk of bleeding in the external validation of the BEST-J score in China. 2. The patient conditions varied. The Japanese study population had a higher average age, more complex medication history and more concomitant diseases. As a result, the number of patients with BEST-J score ≥ 6 was 0, which shows in additional file 1 and additional file 2, leading the AUC of BEST-J in Chinese validation cohort is only 0.624. While the Chinese study population had larger average tumor size, poorer tumor differentiation and more multiple tumors, which increased the difficulty of the ESD operation. As a result, the ESD procedure time was longer and more lesions were resected piecemeal. This also contributed to the higher delayed bleeding rate in the EGC patients in our study (6.1%) than in patients in Japan (4.7%-5.0%).

The previously reported incidence of delayed bleeding ranged from 2.4 to 18% [21, 34–37]. If the endoscopist cannot detect this bleeding in time, some cases may progress to hemorrhagic shock or even death. Therefore, it is valuable to stratify the risk of delayed bleeding after ESD. Endoscopists should pay enough attention to patients with a higher risk of delayed bleeding, be more cautious and apply prophylactic coagulation to prevent delayed bleeding. In our study, five independent influencing factors were used to establish a risk score model to predict the risk of delayed bleeding after ESD: smoking history, tumor size > 20 mm, intraoperative use of hemoclips, resection of multiple tumors and APTT prolongation. All five factors can be assessed before the end of the ESD procedure. Thus, instant estimation of the risk of delayed bleeding can be accomplished based on this risk prediction model, and the results can guide endoscopists to take appropriate prophylactic measures and monitor high-risk patients.

Some of the limitations of this study must be addressed. First, the derivation cohort of this model was based on a single-center retrospective study, and there were only 27 delayed bleeding events, which may pose potential unknown bias and reduce the statistical power. In addition, this also limits our assessment of several previously reported risk factors for delayed bleeding after gastric ESD, such as intraoperative bleeding [38]. Second, the number of patients using anticoagulant and antiplatelet medication was very small. As a result, the effects of these drugs on delayed bleeding were not fully evaluated. Third, the observation time was not long enough. Our observation period was only 2 weeks, but it was reported that delayed bleeding occurred 34 days after ESD for gastric tumors [39]. Finally, the new risk prediction model lacked external validation. Although the rate of delayed bleeding in our study (6.1%) was similar to that of a large meta-analysis (5.8%), it is still not clear whether this model applies to other geographical regions [40]. Despite these limitations, which may undermine the potential for the generalization of our scoring system, our study is valuable since it proposes a useful risk scoring model that incorporates easily assessable clinical components. In fact, external validation of this model is underway, and it is believed that this model can be appropriately applied to other ESD centers in China.

## Conclusions

A history of smoking, tumor size > 20 mm, intraoperative use of hemoclips, resection of multiple tumors, and prolongation of APTT were independent influencing factors of delayed bleeding after ESD for EGC. The risk prediction model including these factors can quantitatively anticipate the risk of delayed bleeding. Immediate estimation of the risk of delayed bleeding with this prediction model may help endoscopists plan ESD procedures, minimize the risk of bleeding after ESD and monitor patients after ESD.

## Supplementary Information

**Additional file 1: **Data comparison between the derivation cohort and the validation cohort.

**Additional file 2: **Comparison of delayed bleeding rate between the derivation cohort and the validation cohort.

## Data Availability

All data generated or analysed during this study are included in this published article and its supplementary information files.

## Abbreviations

APTT: Activated partial thromboplastin time
AUC: Area under the curve
BEST-J: Bleeding after ESD trend from Japan
CI: Confidence interval
CKD: Chronic kidney disease
DOAC: Direct oral anticoagulant
EGC: Early gastric cancer
ESD: Endoscopic submucosal dissection
HGIN: High-grade intraepithelial neoplasia
INR: International standardized ratio
LGIN: Low-grade intraepithelial neoplasia
M: Mucosa
OR: Odds ratio
P2Y12RA: P2Y12 receptor antagonist
PT: Prothrombin time
PT(A): Prothrombin time activity
ROC: Receiver operating characteristic
SM1: Submucosal invasion < 500 µm from the muscularis mucosa
SM2: Submucosal invasion ≥ 500 µm from the muscularis mucosa

## References

[1] Venerito M, Vasapolli R, Rokkas T, Malfertheiner P (2018). Gastric cancer: epidemiology, prevention, and therapy. Helicobacter.

[2] Hu J, Zhao Y, Ren M, Li Y, Lu X, Lu G, Zhang D, Chu D, He S (2018). The comparison between endoscopic submucosal dissection and surgery in gastric cancer: a systematic review and meta-analysis. Gastroenterol Res Practice.

[3] Li Z, Lv B, Lv N, Wang G (2020). Chinese consensus on management of gastric mucosal precancerous conditions and lesions. Chin J Digest Endosc.

[4] Fujishiro M, Yoshida S, Matsuda R, Narita A, Yamashita H, Seto Y (2017). Updated evidence on endoscopic resection of early gastric cancer from Japan. Gastric Cancer.

[5] Oda I, Suzuki H, Nonaka S, Yoshinaga S (2013). Complications of gastric endoscopic submucosal dissection. Digest Endosc.

[6] van Bussel EF, Richard E, Busschers WB, Steyerberg EW, van Gool WA, Moll van Charante EP, Hoevenaar-Blom MP (2019). A cardiovascular risk prediction model for older people: Development and validation in a primary care population. J Clin Hypertens (Greenwich Conn)..

[7] Kim SM, Lee H, Min BH, Kim JJ, An JY, Choi MG, Bae JM, Kim S, Sohn TS, Lee JH (2019). A prediction model for lymph node metastasis in early-stage gastric cancer: toward tailored lymphadenectomy. J Surg Oncol.

[8] Tellum T, Nygaard S, Skovholt EK, Qvigstad E, Lieng M (2018). Development of a clinical prediction model for diagnosing adenomyosis. Fertil Steril.

[9] Hatta W, Tsuji Y, Yoshio T, Kakushima N, Hoteya S, Doyama H, Nagami Y, Hikichi T, Kobayashi M, Morita Y, Sumiyoshi T, Iguchi M, Tomida H, Inoue T, Koike T, Mikami T, Hasatani K, Nishikawa J, Matsumura T, Nebiki H, Nakamatsu D, Ohnita K, Suzuki H, Ueyama H, Hayashi Y, Sugimoto M, Yamaguchi S, Michida T, Yada T, Asahina Y, Narasaka T, Kuribasyashi S, Kiyotoki S, Mabe K, Nakamura T, Nakaya N, Fujishiro M, Masamune A (2020). Prediction model of bleeding after endoscopic submucosal dissection for early gastric cancer: BEST-J score. Gut.

[10] Chai NL, Zhai YQ, Du C (2018). Expert consensus on endoscopic standardized resection of early gastric cancer (2018, Beijing). Chin J Gastrointest Endosc (Electronic Edition).

[11] Kim SG, Park CM, Lee NR, Kim J, Lyu DH, Park SH, Choi IJ, Lee WS, Park SJ, Kim JJ, Kim JH, Lim CH, Cho JY, Kim GH, Lee YC, Jung HY, Lee JH, Chun HJ, Seol SY (2018). Long-term clinical outcomes of endoscopic submucosal dissection in patients with early gastric cancer: a prospective multicenter cohort study. Gut and liver.

[12] Yang CH, Qiu Y, Li X, Shi RH (2020). Bleeding after endoscopic submucosal dissection of gastric lesions. J Dig Dis.

[13] Association JGC (2011). Japanese classification of gastric carcinoma: 3rd English edition. Gastric Cancer.

[14] Ono H, Yao K, Fujishiro M, Oda I, Nimura S, Yahagi N, Iishi H, Oka M, Ajioka Y, Ichinose M, Matsui T (2016). Guidelines for endoscopic submucosal dissection and endoscopic mucosal resection for early gastric cancer. Digest Endosc.

[15] Expert consensus on standardized endoscopic resection of early gastric cancer. Chin J Digest Endosc. 2019;36;381–92.

[16] Organization WH. Guidelines for controlling and monitoring the tobacco epidemic. World Health Organization; 1998

[17] Shi Y, Cui T, Tang Y, Liu J, Tan N (2020). Influence of smoking on the risk of bleeding after coronary intervention. Guangdong Med J.

[18] Nordestgaard AT, Rasmussen LS, Sillesen M, Steinmetz J, King DR, Saillant N, Kaafarani HM, Velmahos GC (2020). Smoking and risk of surgical bleeding: nationwide analysis of 5,452,411 surgical cases. Transfusion.

[19] Powell JT (1998). Vascular damage from smoking: disease mechanisms at the arterial wall. Vasc Med (London, England).

[20] Sørensen LT (2012). Wound healing and infection in surgery: the pathophysiological impact of smoking, smoking cessation, and nicotine replacement therapy: a systematic review. Ann Surg.

[21] Lan S, Huang M, Lin D, Chen L (2020). Study on the efficacy of endoscopic submucosal dissection in the treatment of early gastric cancer and the related factors of postoperative bleeding. J Pract Med Tech.

[22] Zhao Y, Xu G, Lv Y, Zhang X, Ling T, Zhang Y, Wang L, Zou X (2017). Risk factors of postoperative bleeding in elderly patients after endoscopic submucosal dissection for early gastric neoplasm. Pract Geriatr.

[23] Libânio D, Costa MN, Pimentel-Nunes P, Dinis-Ribeiro M (2016). Risk factors for bleeding after gastric endoscopic submucosal dissection: a systematic review and meta-analysis. Gastrointest Endosc.

[24] Guo Z, Miao L, Chen L, Hao H, Xin Y (2018). Efficacy of second-look endoscopy in preventing delayed bleeding after endoscopic submucosal dissection of early gastric cancer. Exp Ther Med.

[25] Turan AS, Ultee G, Van Geenen EJM, Siersema PD (2019). Clips for managing perforation and bleeding after colorectal endoscopic mucosal resection. Expert Rev Med Devices.

[26] Zhang QS, Han B, Xu JH, Gao P, Shen YC (2015). Clip closure of defect after endoscopic resection in patients with larger colorectal tumors decreased the adverse events. Gastrointest Endosc.

[27] Liu T, Hua C, Hu W, Wang G (2021). Effect of preventive hemostatic clip on preventing delayed bleeding after gastric polypectomy. China Med Herald.

[28] Azumi M, Takeuchi M, Koseki Y, Kumagai M, Kobayashi Y, Takatsuna M, Yoshioka A, Yoshikawa S, Miura T, Terai S (2019). The search, coagulation, and clipping (SCC) method prevents delayed bleeding after gastric endoscopic submucosal dissection. Gastric Cancer.

[29] Zhang G, Meng J, Fan X (2007). Significance of Blood Coagulation function in preventing Hemorrhage in premature infants. Chin J Child Health Care.

[30] Hasegawa M, Wada H, Tone S, Yamaguchi T, Wakabayashi H, Ikejiri M, Watanabe M, Fujimoto N, Matsumoto T, Ohishi K, Yamashita Y, Katayama N, Sudo A (2018). Monitoring of hemostatic abnormalities in major orthopedic surgery patients treated with edoxaban by APTT waveform. Int J Lab Hematol.

[31] Xia C, Zhuo W, Dan Y, Yu-ming W, Ying H, Run M, Ming-liang L (2020). APTT ACTIN FSL reagent was used in clinical laboratory to establish activation partial thrombin time reference range. Chin J Thrombosis Hemostasis.

[32] Kazuhide H, Mototsugu K, Seiji H, Kazuma F, Masahiro I, Kazunari M, Noriya U (2018). Guidelines for gastroenterological endoscopy in patients undergoing antithrombotic treatment: 2017 appendix? on anticoagulants including direct oral anticoagulants. Digest Endosc.

[33] Li Z, Linghu E, Wang L (2020). Chinese expert consensus on ESD-related adverse events (2020, Wuxi). Chin J Digest Endosc.

[34] Fu Q. Analysis of clinical risk factors of postoperative bleeding and perforation during endoscopic submucosal dissection. Qing Dao University. 2017

[35] Nam HS, Choi CW, Kim SJ, Kim HW, Kang DH, Park SB, Ryu DG (2019). Risk factors for delayed bleeding by onset time after endoscopic submucosal dissection for gastric neoplasm. Sci Rep.

[36] Sanomura Y, Oka S, Tanaka S, Yorita N, Kuroki K, Kurihara M, Mizumoto T, Yoshifuku Y, Chayama K (2018). Taking warfarin with heparin replacement and direct oral anticoagulant is a risk factor for bleeding after endoscopic submucosal dissection for early gastric cancer. Digestion.

[37] Shindo Y, Matsumoto S, Miyatani H, Yoshida Y, Mashima H (2016). Risk factors for postoperative bleeding after gastric endoscopic submucosal dissection in patients under antithrombotics. World J Gastrointest Endosc.

[38] Huang Z, Deng H, Jia Y (2017). Analysis of risk factors of delayed bleeding after ESD for early gastric cancer and nursing countermeasures. Med J Natl Defend Forces Southwest China.

[39] Okada K, Yamamoto Y, Kasuga A, Omae M, Kubota M, Hirasawa T, Ishiyama A, Chino A, Tsuchida T, Fujisaki J, Nakajima A, Hoshino E, Igarashi M (2011). Risk factors for delayed bleeding after endoscopic submucosal dissection for gastric neoplasm. Surg Endosc.

[40] Zullo A, Manta R, De Francesco V, Manfredi G, Buscarini E, Fiorini G, Vaira D, Marmo R. Endoscopic submucosal dissection of gastric neoplastic lesions in Western countries: systematic review and meta-analysis. Eur J Gastroenterol Hepatol. 2020 10.1097/MEG.0000000000001886 32804845

