# Supplementary material for: External validation of the BEST-J score and a new risk prediction model for ESD delayed bleeding in patients with early gastric cancer
Source: BMC Gastroenterol. 2022 Apr 20;22:194. doi: 10.1186/s12876-022-02273-2 (PMC9022319; doi:10.1186/s12876-022-02273-2)
Supplement: Supplementary file 1 — Additional file 1: Data comparison between the derivation cohort and the validation cohort. [file 12876_2022_2273_MOESM1_ESM.docx]

Supplementary Table 1 Data comparison between the derivation cohort and the validation cohort

|  | Derivation cohort (n=8291) | Validation cohort (n=444) | χ^2^ or t value | *P* value |
| --- | --- | --- | --- | --- |
| Age (years，mean±SD) | 68.51±1.48 | 63.07±9.50 | 12.053 | <0.001** |
| Age [n (%)] |  |  | 140.585 | <0.001** |
| ≥75 years old | 3278 (39.5) | 51 (11.5) |  |  |
| <75 years old | 5013 (60.5) | 393 (88.5) |  |  |
| Sex [n (%)] |  |  | 3.656 | 0.056 |
| Male | 6182 (74.6) | 313 (70.5) |  |  |
| Female | 2109 (25.4) | 131 (29.5) |  |  |
| Warfarin [n (%)] |  |  | 10.171 | 0.001* |
| Yes | 255 (3.1) | 2 (0.5) |  |  |
| No | 8036 (96.9) | 442 (99.5) |  |  |
| Cilostazol [n (%)] |  |  | 9.173 | 0.002* |
| Yes | 168 (2.0) | 0 (0.0) |  |  |
| No | 8123 (98.0) | 444 (100.0) |  |  |
| P2Y12RA [n (%)] |  |  | 0.317 | 0.573 |
| Yes | 344 (4.1) | 16 (3.6) |  |  |
| No | 7947 (95.9) | 428 (96.4) |  |  |
| Aspirin [n (%)] |  |  | 5.254 | 0.022* |
| Yes | 793 (9.6) | 28 (6.3) |  |  |
| No | 7498 (90.4) | 416 (93.7) |  |  |
| DOAC [n (%)] |  |  | 8.359 | 0.004* |
| Yes | 189 (2.3) | 1 (0.2) |  |  |
| No | 8102 (97.7) | 443 (99.8) |  |  |
| Interruption of AT agents^†^ [n (%)] | |  | 4.129 | 0.042* |
| Yes | 1077 (13.0) | 43 (9.7) |  |  |
| No | 7211 (87.0) | 401 (90.3) |  |  |
| ESD procedure time^†^ [n (%)] |  |  | 3.898 | 0.048* |
| >120 min | 1447 (17.5) | 94 (21.2) |  |  |
| ≤120 min | 6821 (82.5) | 350 (78.8) |  |  |
| Resection type [n (%)] |  |  | 105.035 | <0.001** |
| En bloc | 8243 (99.4) | 421 (94.8) |  |  |
| Piecemeal | 48 (0.6) | 23 (5.2) |  |  |
| Tumor size (mm, mean±SD) | 15.35±9.63 | 18.38±11.02 | 5.675 | <0.001** |
| Tumor size >30 mm [n (%)] |  |  | 0.370 | 0.543 |
| Yes | 956 (11.5) | 47 (10.6) |  |  |
| No | 7335 (88.5) | 397 (89.4) |  |  |
| Tumor location [n (%)] |  |  | 53.460 | <0.001** |
| Lower third | 3811 (46.0) | 283 (63.7) |  |  |
| Other location | 4480 (54.0) | 161 (36.3) |  |  |
| Multiple tumors [n (%)] |  |  | 10.087 | 0.001** |
| Yes | 1014 (12.2) | 32 (7.2) |  |  |
| No | 7277 (87.8) | 412 (92.8) |  |  |
| Tumor differentiation [n (%)] |  |  | 9.399 | 0.002** |
| Differentiated | 7846 (94.6) | 405 (91.2) |  |  |
| Undifferentiated | 445 (5.4) | 39 (8.8) |  |  |
| CKD with hemodialysis [n (%)] | |  | 7.012 | 0.008** |
| Yes | 129 (1.6) | 0 (0.0) |  |  |
| No | 8162 (98.4) | 444 (100.0) |  |  |
| BEST-J score [n (%)]^†^ |  |  | NA | <0.001** |
| 0 | 2923 (35.3) | 26 (5.9) |  |  |
| 1 | 3344 (40.3) | 194 (43.7) |  |  |
| 2 | 1059 (12.8) | 185 (41.7) |  |  |
| 3 | 471 (5.7) | 31 (7.0) |  |  |
| 4 | 289 (3.5) | 6 (1.4) |  |  |
| 5 | 123 (1.5) | 2 (0.5) |  |  |
| 6 | 53 (0.6) | 0 (0.0) |  |  |
| 7 | 16 (0.2) | 0 (0.0) |  |  |
| 8 | 10 (0.1) | 0 (0.0) |  |  |
| BEST-J risk category [n (%)]^†^ | |  | 292.863 | <0.001** |
| Low risk | 6267 (75.6) | 220 (49.5) |  |  |
| Intermediate risk | 1059 (12.8) | 185 (41.7) |  |  |
| High risk | 760 (9.2) | 37 (8.3) |  |  |
| Very high risk | 202 (2.4) | 2 (0.5) |  |  |
| BEST-J score (points, mean±SD) | 1.10±1.24 | 1.56±0.80 | -7.591 | <0.001** |

AT, antithrombotic; BEST-J, bleeding after ESD trend from Japan; CKD, chronic kidney disease; DOAC, direct oral anticoagulant; EGC, early gastric cancer; ESD, endoscopic submucosal dissection; NA, not available due to Fisher’s exact test; P2Y12RA, P2Y12 receptor antagonist; SD, standard deviation.

^†^There were missing data (3 cases in interruption of AT agents and 23 cases in ESD procedure time) in the derivation cohort.

*P<0.05, **P<0.01
