# Supplementary material for: External validation of the BEST-J score and a new risk prediction model for ESD delayed bleeding in patients with early gastric cancer
Source: BMC Gastroenterol. 2022 Apr 20;22:194. doi: 10.1186/s12876-022-02273-2 (PMC9022319; doi:10.1186/s12876-022-02273-2)
Supplement: Supplementary file 2 — Additional file 2: Comparison of delayed bleeding rate between the derivation cohort and the validation cohort. [file 12876_2022_2273_MOESM2_ESM.docx]

Supplementary Table 2 Comparison of delayed bleeding rate between the derivation cohort and the validation cohort

|  | Derivation cohort (n/N, %) | Validation cohort (n/N, %) | χ^2^ value | *P* value |
| --- | --- | --- | --- | --- |
| Total delayed bleeding rate | 387/8288^†^ (4.7) | 27/444 (6.1) | 1.860 | 0.173 |
| BEST-J score |  |  |  |  |
| 0 | 58/2923 (2.0) | 1/26 (3.8) | <0.001 | 0.410 |
| 1 | 117/3344 (3.5) | 7/194 (3.6) | 0.006 | 0.936 |
| 2 | 65/1059 (6.1) | 14/185 (7.6) | 0.541 | 0.462 |
| 3 | 45/471 (9.6) | 3/31 (9.7) | <0.001 | 1.000 |
| 4 | 42/289 (14.5) | 1/6 (16.7) | <0.001 | 1.000 |
| 5 | 26/123 (21.1) | 1/2 (50.0) | 0.014 | 0.387 |
| 6 | 22/53 (41.5) | 0 (0.0) |  | - |
| 7 | 7/16 (43.8 | 0 (0.0) |  | - |
| 8 | 5/10 (50.0) | 0 (0.0) |  | - |
| BEST-J risk category | |  |  |  |
| Low-risk | 175/6267 (2.8) | 8/220 (3.6) | 0.552 | 0.457 |
| Intermediate-risk | 65/1059 (6.1) | 14/185 (7.6) | 0.541 | 0.462 |
| High-risk | 87/760 (11.4) | 4/37 (10.8) | NA | 1.000 |
| Very high-risk | 60/202 (29.7) | 1/2 (50.0) | NA | 0.510 |

BEST-J, bleeding after ESD trend from Japan; NA, not available due to Fisher’s exact test.

^†^There were data missing from three cases in the derivation cohort.
